# Vitamin D receptor gene polymorphism in oral cancer as a function of tobacco consumption: an evidence based systematic review and meta-analysis

**DOI:** 10.3389/froh.2025.1550683

**Published:** 2025-04-28

**Authors:** Tribikram Debata, Amrita Swain, Soumya Ranjan Jena, Surya Narayan Das, Niranjan Mishra, Luna Samanta

**Affiliations:** ^1^ Department of Oral and Maxillofacial Pathology, SCB Dental College and Hospital, Cuttack, Odisha, India; ^2^ Redox Biology and Proteomics Laboratory, Department of Zoology, Ravenshaw University, Cuttack, India; ^3^ Centre of Excellence for Environment & Public Health, Ravenshaw University, Cuttack, India; ^4^ Department of Oral and Maxillofacial Surgery, SCB Dental College and Hospital, Cuttack, Odisha, India

**Keywords:** VDR gene, vitamin D, tobacco, oral cancer, oral lichen planus

## Abstract

**Background:**

The association between Vitamin D Receptor (VDR) polymorphisms and different cancers has attracted growing attention; nonetheless, the function of these genetic variants in tobacco-related oral cancer remains little comprehended. This review assesses and integrates research concerning the influence of VDR gene variants on the development of tobacco-related oral cancer, emphasizing genetic underpinnings of individual vulnerability and possible tailored preventative approaches.

**Materials and methods:**

The search strategy for this systematic review and meta-analysis was devised to comprehensively identify relevant studies from diverse sources. The investigation included three primary components: the VDR gene, oral cancer, and tobacco. The data from the papers included in the study were independently retrieved by two reviewers. The incidence was evaluated as an odds ratio (OR) with 95% confidence interval (95% CI) using SPSS software.

**Results:**

A preliminary search of biomedical electronic research databases (PubMed, Web of Science, Scopus, Embase, and the Cochrane Library) yielded 60,345 papers. After multi-phase exclusions, five studies met the inclusion criteria. The meta-analysis highlights interactions between genetic polymorphisms, smoking, aging, and oral health risks. The CYP24A1 (rs2296241) heterozygote genotype significantly reduces oral cancer risk (OR = 0.281, *P* = 0.00001). Variants rs1544410 and rs2228570 influence oral health outcomes. The rs2239185 TT (OR = 2.68, *P* = 0.009) and rs7975232 CC (OR = 2.25, *P* = 0.026) increase oral lichen planus risk. Older age is significantly linked to OSCC risk (*P* = 0.001).

**Conclusion:**

This research underscores the role of VDR gene variants in tobacco-related oral cancer. Further studies are essential to validate findings and explore underlying mechanisms.

**Systematic Review Registration:**

https://www.crd.york.ac.uk/PROSPERO/view/CRD42024587292, identifier: CRD42024587292.

## 1 Introduction

Oral cancer, particularly oral squamous cell carcinoma (OSCC), poses a significant global health challenge, with tobacco use identified as the primary risk factor for its development (1). Tobacco exposure, whether through smoking or chewing, introduces carcinogens to the oral mucosa, leading to genetic mutations, chronic inflammation, and cellular damage, which can ultimately result in cancer. However, not everyone exposed to tobacco develops oral cancer, suggesting that genetic factors may play a role in susceptibility (2). Vitamin D has been linked to anti-tumor effects and a reduced risk of several malignancies, including squamous cell carcinoma of the head and neck, when consumed in adequate amounts (3).

**Figure 1:**
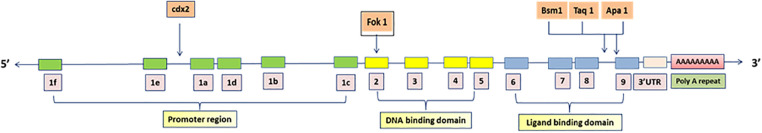
Single nucleotide polymorphic sites in VDR gene. Over 200 variations, including restriction fragment length polymorphisms (RFLPs) and variable number tandem repeats (VNTRs), have been identified in the DNA sequences of the VDR gene. The VDR gene, located on the long arm of chromosome 12, was initially thought to have 9 exons (1–9). Then two additional exons upstream of the previously known exon 1 were identified and name 1a and 1b and original exon 1 was renamed exon 1c with the translation start codon in exon 2. All down stream exons were numbered in roman numerals.

Vitamin D is a steroid hormone produced in epidermal keratinocytes in response to UVB light (290–315 nm) or obtained through dietary sources. It undergoes two hydroxylation steps—25-hydroxylation and 1-*α*-hydroxylation—to become the active hormone 1,25-dihydroxyvitamin D. This hormone binds to its nuclear receptor, the vitamin D receptor (VDR), in target tissues, inducing conformational changes that promote heterodimerization with the retinoid X receptor (RXR) and zinc finger-mediated binding to vitamin D response elements (VDREs) in the regulatory regions of target genes (4)**.** The human VDR gene is located on chromosome 12q13.11, comprising 14 exons that span approximately 64 kbp of DNA. The VDR protein can consist of either 427 or 424 amino acids, depending on a T to C polymorphism (ATG to ACG) at the translational start site (5). Key single-nucleotide polymorphisms (SNVs) in the VDR gene include *FokI*C > T (rs2228570), *BsmI*A > G (rs1544410), *ApaI*G > T (rs7975232), *TaqI*C > T (rs731236), and *Cdx-2*(rs11568820) (6) (Figure 1).

**Figure 2:**
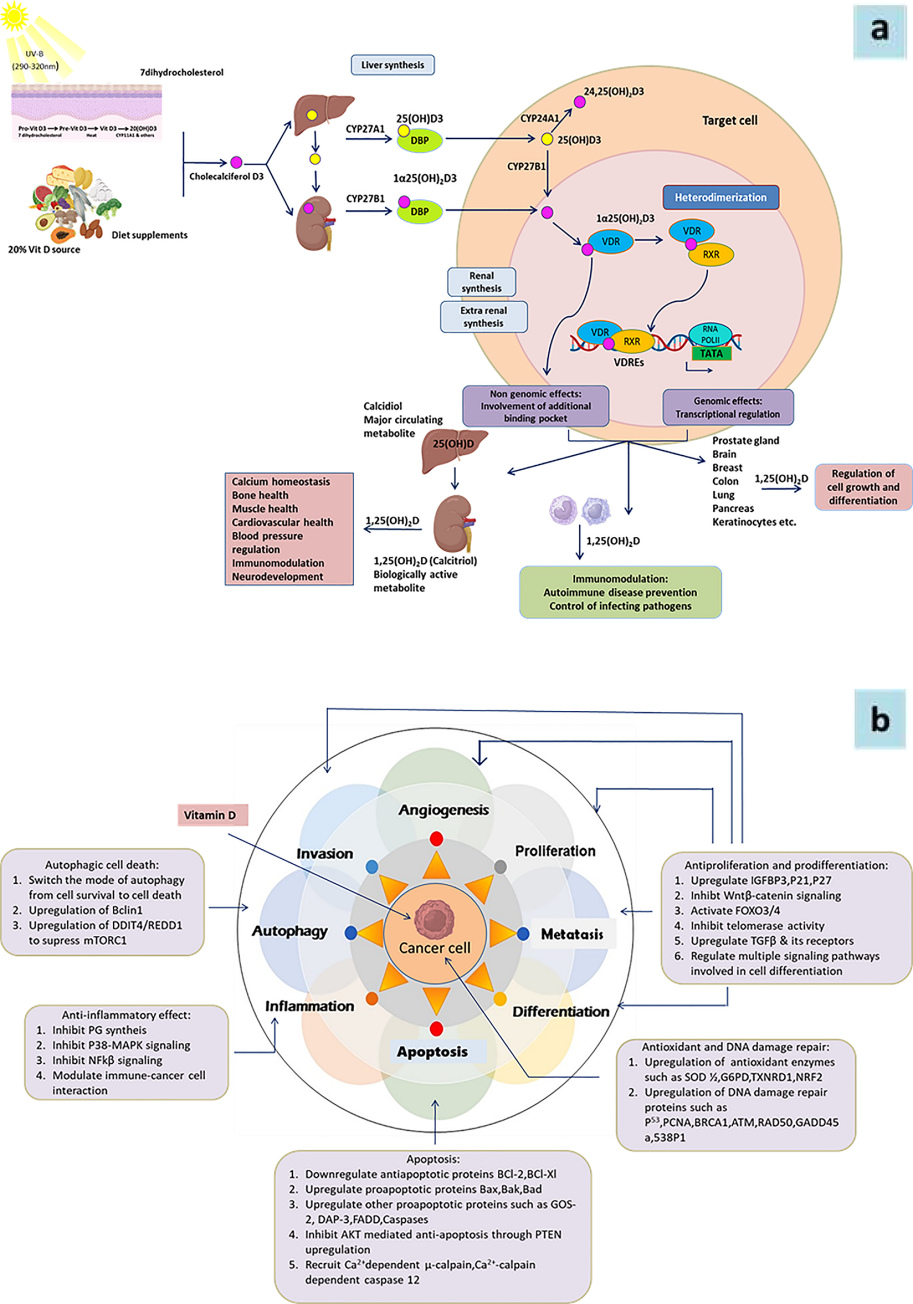
**(a)** Schematic representation of vitamin D metabolism and its biological functions in target cell. Vitamin D is derived from dietary sources or synthesized in the skin from 7-dehydrocholesterol (7-DHC) upon sunlight exposure. In the liver, vitamin D3 undergoes hydroxylation to form 25-hydroxyvitamin D3 [25(OH)D3], which is further hydroxylated in the kidney and other tissues, such as immune and epithelial cells, to produce the biologically active form, 1*α*,25-dihydroxyvitamin D3 [1*α*,25(OH)₂D3]. In circulation, vitamin D metabolites are transported by vitamin D-binding protein (VDBP). The active form, 1*α*,25(OH)₂D3, enters cells and binds to the vitamin D receptor (VDR), facilitating the formation of the VDR-retinoid X receptor (VDR-RXR) complex. This complex translocates to the nucleus and interacts with vitamin D response elements (VDREs) to regulate gene expression. Vitamin D exerts diverse biological functions across multiple organs and tissues. Its active metabolites are eventually degraded in the kidney and other target tissues and excreted in the urine. 7DHC, 7-dehydrocholesterol; VDBP, vitamin D binding protein; VDR, vitamin D receptor; RXR, retinoid X receptor; VDRE, vitamin D response element. **(b)** Role of Vitamine D in differentiation, proliferation, invation and metastasis of cancer.

Given that about 3% of the human genome is regulated by the vitamin D endocrine system (7), it is clear that vitamin D plays a significant role in regulating cellular functions (Figure 2a). Its involvement in cell cycle regulation is evidenced by the expression of p21 and p27 (cyclin-dependent kinase inhibitors that facilitate cell cycle arrest), and the receptor is also essential for regulating calcium and phosphate balance, skeletal metabolism, and interactions with retinoid signaling and fibronectin pathways (8, 9). Therefore, its significance in modulating tumor microenvironment and consequently cancer cannot be ruled out (Figure 2b).

Significant associations have been identified between VDR polymorphisms and various cancers, including prostate (*Fok1, Bsm1, Taq1*), breast (*Fok1, Bsm1, Taq1*), colon-rectum (*Fok1, Bsm1, Taq1*), and cutaneouscancer (*Fok1, Bsm1, Taq1*). However, few studies have reported risk estimates for other cancer types. Notably, a recent study by Mohtasham et al. (2024) found an association between the *ApaI* SNV and OSCC^,^ suggesting that VDR polymorphism could serve as an important biomarker for assessing the risk of developing OSCC (10).

A meta-analysis that searched MEDLINE and ResearchGate up to June 2017 identified 12 articles covering 26 studies on VDR polymorphisms (*FokI, ApaI, TaqI, BsmI*) related to tobacco-associated lung, neck, head, esophageal, and oral cancers. This analysis included 5,113 cases and 5,657 controls, revealing a significant association between the *TaqI* polymorphism and the risk of tobacco-related cancers (11). Additionally, the A allele of *ApaI*(rs7975232) in the VDR gene, in conjunction with its interaction with smoking, was linked to an increased risk of renal cell carcinoma (12). Given this background, this systematic review aims to evaluate and synthesize evidence regarding the role of VDR gene polymorphisms in the development of tobacco-associated oral cancerby analyzing the existing evidence, emphasizing the genetic mechanisms underlying individual susceptibility and potential personalized prevention strategies.

## 2 Material and methods

The systematic review and meta-analysis were registered in PROSPERO (Registration Number: CRD42024587292) on September 17, 2024, prior to the commencement of the study.

### 2.1 Study design

This systematic review adhered to the PRISMA (Preferred Reporting Items for Systematic Reviews and Meta-Analyses) standards. The aim was to evaluate the correlation (if any) between Vitamin D receptor (VDR) gene variation with tobacco-related oral cancer by analyzing the existing evidence.

### 2.2 The PICO framework for evidence synthesis

PICO stands for patient/population, intervention, comparison and outcomes. This framework helps the investigator to formulate focused research questions in systematic reviews and meta-analysis, by structuring them around Patient/Population, Intervention, Comparison/Control, and Outcome. The question for the current study was framed using PubMed PICO Tool [https://www.nlm.nih.gov/oet/ed/pubmed/pubmed_in_ebp/02-100.html (13, 14)].

P (Population): Patients with tobacco-associated oral cancer; I (Intervention/Exposure): Vitamin D receptor (VDR) gene polymorphisms (specific variants such as *FokI, BsmI, TaqI, ApaI* etc.); C (Comparison): Patients without, VDR gene polymorphisms or individuals with different VDR genotypes (e.g., healthy individuals, non-tobacco users, or those with other genetic polymorphisms); O (Outcome): Risk, incidence, and progression of oral cancer in relation to tobacco use and VDR gene polymorphisms.

### 2.3 Search strategy

The search method for this systematic review was devised to thoroughly locate pertinent studies from various sources. We examined five important electronic databases: PubMed, Web of Science, Scopus, Embase, and the Cochrane Library. The search criteria were formulated using a blend of Medical Subject Headings (MeSH) and free-text keywords to encompass all pertinent research examining the correlation between VDR gene polymorphisms and oral cancer, especially concerning tobacco consumption.

The investigation encompassed three key elements: VDR gene, oral cancer, and tobacco. Keywords associated with the VDR gene comprised “Vitamin D receptor”, “VDR”, “VDR gene,” and “Vitamin D receptor polymorphism”. These phrases were amalgamated with cancer-related terminology including “oral cancer”, “oral squamous cell carcinoma” and “oral malignancies.” To encompass the tobacco-related dimension, terminology such as “tobacco,” “smoking,” “chewing tobacco,” and “tobacco use” were incorporated. Boolean operators such as AND and OR were employed to amalgamate these terms, guaranteeing the inclusion of all pertinent research examining the correlation between VDR gene polymorphisms and tobacco-related oral cancer.

Filters were implemented to limit findings to human research and publications in English. Furthermore, thorough examinations of the reference lists of the included research were performed to uncover additional pertinent papers. This search approach was developed to be exhaustive, guaranteeing the incorporation of all pertinent research regarding the influence of VDR gene polymorphisms on the progression of tobacco-related oral cancer.

### 2.4 Inclusion criteria

Human studies (clinical trials, case-control studies, cohort studies, cross-sectional studies); Studies examining VDR gene polymorphisms in patients with tobacco-associated oral cancer; Articles published in English; Studies with clear data on the relationship between VDR polymorphisms and oral cancer risk.

### 2.5 Exclusion criteria

Non-human studies or animal models; Studies without genetic analysis of VDR polymorphisms; Studies without a focus on tobacco-related oral cancer; Reviews, editorials, case reports, and conference abstracts.

### 2.6 Study selection and data extraction

Two independent reviewers assessed the subject matter and outlines of all extracted studies. The suitability of full-text materials was assessed based on the defined inclusion and exclusion criteria. Inconsistencies among reviewers were resolved through deliberation, and when consensus could not be reached, a third reviewer was checked out. Data from the studies included in the analysis were extracted independently by two reviewers.

### 2.7 Quality assessment

The risk of bias for the case-control studies in this systematic review was evaluated using the Newcastle-Ottawa Scale (NOS), which assesses research across three domains: Selection, Comparability, and Exposure. In the Selection domain, studies were evaluated based on the representativeness of cases, the suitable selection of controls, and the reliability of procedures for diagnosing oral cancer and testing VDR gene polymorphisms. In the Comparability domain, points were allocated for managing significant confounders, notably tobacco consumption, along with additional characteristics such as age, gender, and lifestyle. The Exposure domain evaluated the determination of VDR polymorphism genotyping, methodological consistency between cases and controls, and rates of non-response. Studies that received scores of 7 or more stars (out of 9) were classified as low risk of bias, whilst those getting fewer than 5 stars were deemed high risk. Discrepancies in score were addressed through discussion or consultation with a third reviewer to ensure precision and mitigate bias in the review process.

### 2.8 Meta-analysis

Due to availability of limited number of studies (*n* = 05), publication bias is not assessed. The risk of bias for each study was assessed using the Newcastle-Ottawa Quality Assessment Scale for case-control studies, which evaluates three main domains: selection, comparability, and outcome, with a total score of nine points. To assess heterogeneity across studies, the I² test was applied.

### 2.9 Statistical analysis

The data were analyzed using SPSS version 29. A two-tailed *P* value below 0.05 was considered statistically significant. For the forest plot analysis, the odds ratio with a 95% confidence interval was used as the measure of effect.

## 3 Results

### 3.1 Study selection and characteristics

**Figure 3:**
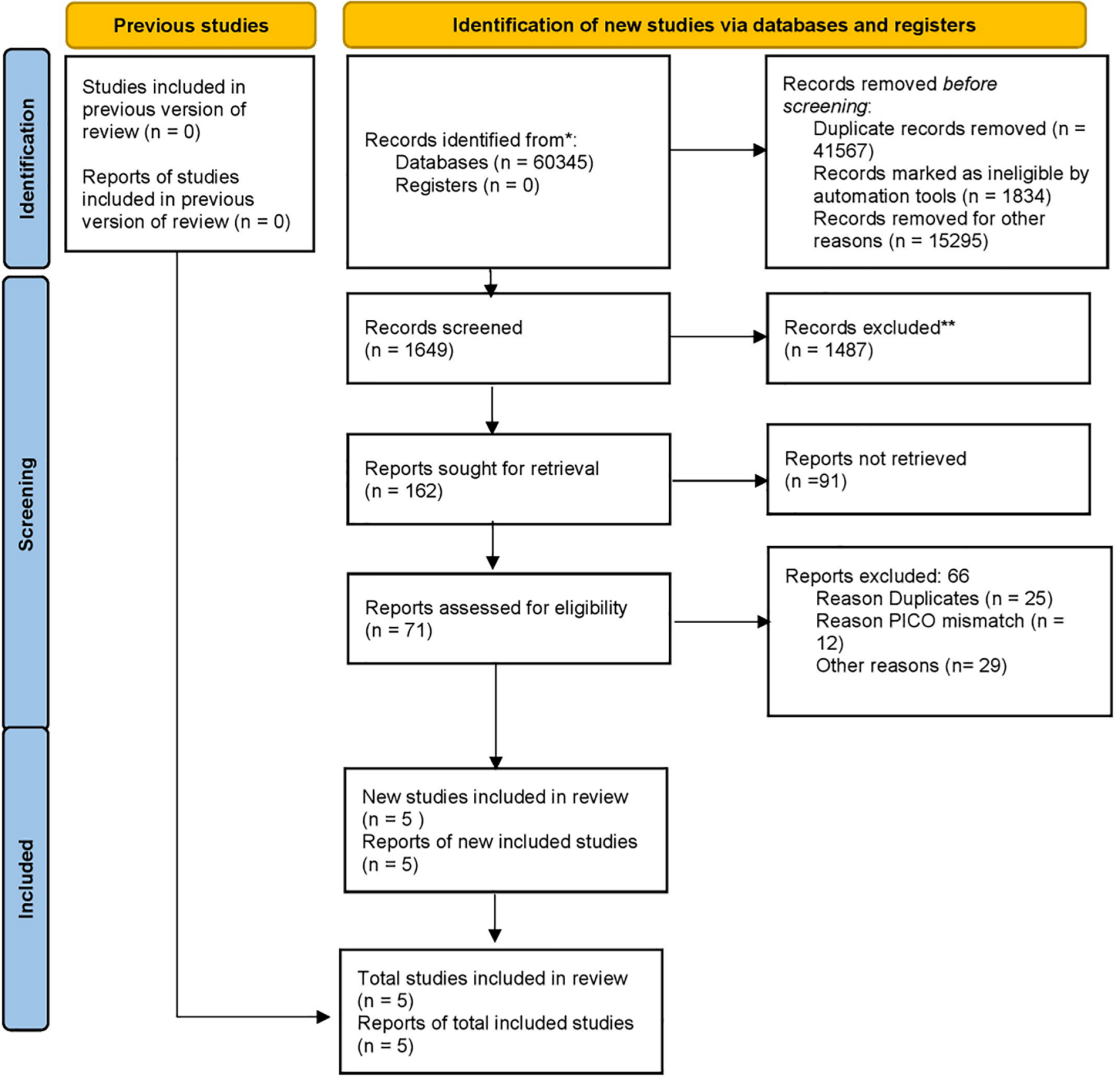
PRISMA 2020 flow diagram illustrating the number of records included and excluded at various screening and revieweing steps, leading to final list of records for data extraction and meta-analysis.

**Figure 4:**
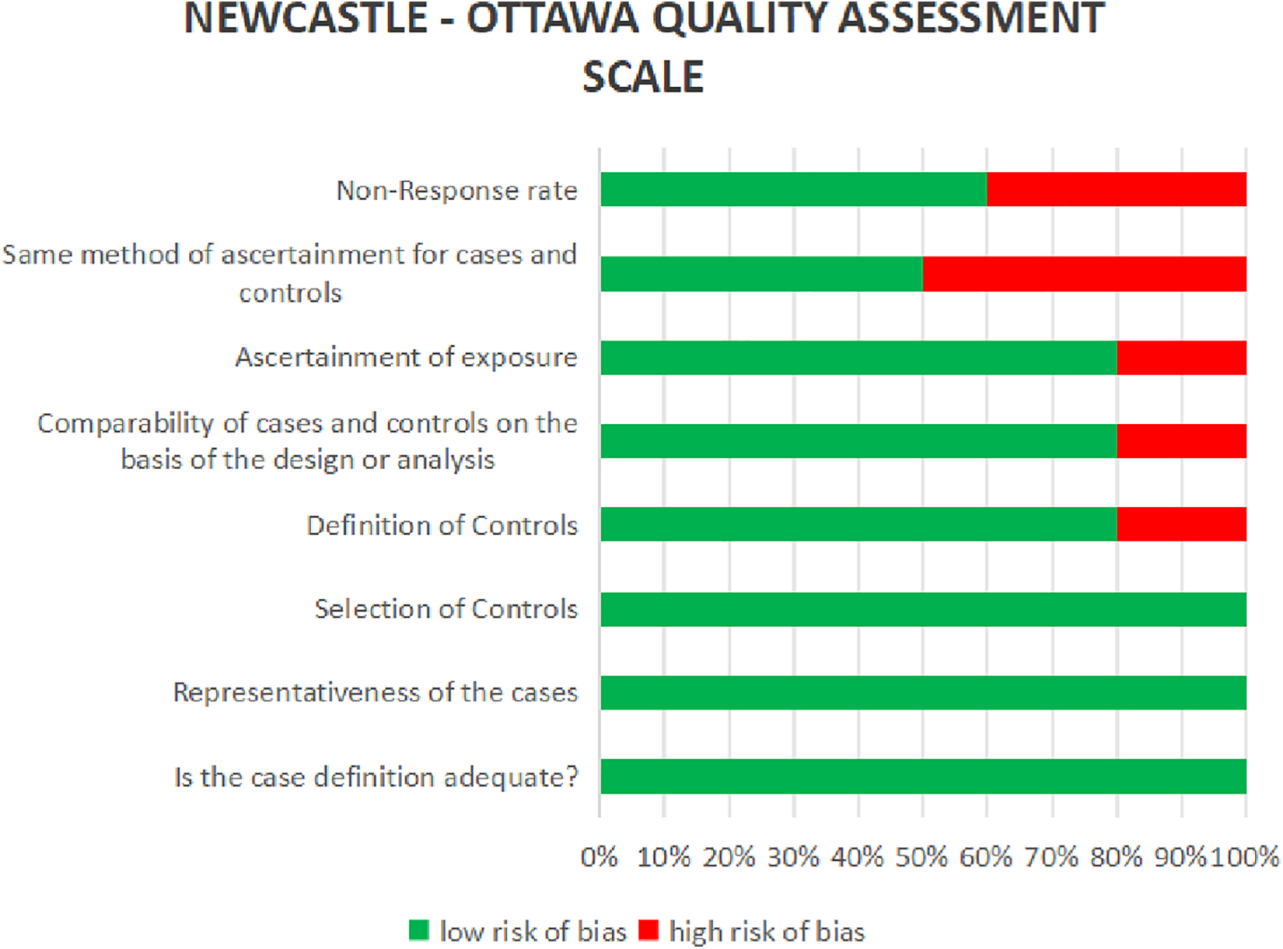
Risk of bias assessment based on the Newcastle–Ottawa scale of studies included in this systematic review analysis.

The process of doing this systematic review started with an initial search of computer databases, which yielded a total of 6345 documents. Following the elimination of 41,567 duplicate entries, there were a total of 1,649 unique records that were left for analysis. After evaluating the titles and abstracts of 1487 records, it was determined that they did not meet the inclusion requirements or were irrelevant to the study subject. As a result, these records were no longer considered for inclusion. There were a total of 162 records that were submitted for full-text retrieval, and out of them 71 records were successfully obtained. As a result of the evaluation of these full-text papers, 66 studies were disregarded for a variety of reasons, including PICO inconsistencies and extra considerations. The final synthesis consisted of five studies that were able to satisfy all of the inclusion criteria and were therefore included (Figure 3). The data extraction from all included studies is presented in Table 1. The quality assessment of the included studies indicated that the majority exhibited a low risk of bias across nearly all categories of the Newcastle-Ottawa Scale (Figure 4).

**Table 1:** Case control studies (*n* = 5) showing effect of VDR polymorphisms on the risk of tobacco-related cancers.

Sl. No.	Author, Year (Ref no.)	Country	Sample size	SNVs analysed	Genotyping method used	Key findings	Association with oral cancer
1	Zeljic et al. (15)	Serbia	110-Cases	s4516035 (EcoRV), rs2228570 (FokI), rs731236 (TaqI) and rs7975232 (ApaI) (rs1544410 (BsmI) and rs11574085 (VDR) polymor phisms in VDR gene as well as SNVs in genes involved in vitamin D metabolism rs464653 (CYP27B) and rs2296241 (CYP24A1)	PCR–RFLP or real-time PCR SNV analysis method.	Polymorphism in the CYP24A1gene may affect susceptibility to oral cancer. The VDR FokI polymorphism was linked to poorer survival and may serve as an independent prognostic indicator.	A notable reduction in oral cancer risk was seen in persons possessing the heterozygous genotype of the CYP24A1 gene (rs2296241) compared to the wild type.
			122-Control				
2	Suchanecka et al. (16)	Poland	200-Cases	rs7975232 (ApaI), rs1544410 (BsmI) and rs2228570 (Fok1) polymorphisms	PCR	In smokers, there were statistically significant differences in genotype frequencies for the VDR rs1544410 gene compared to the control group, while, there were no significant differences in allele frequencies between smokers and non-smokers. No differences were observed in the frequencies of the rs2228570 and rs7975232 genotypes and alleles between smokers and non-smokers.	The influence of genetic variations, as well as their interaction with smoking on the examined parameters, was evident.
			200-controls				
3	Shen et al. (17)	China	177-Cases	Eight SNVs: rs731236, rs739837, rs757343, rs2107301, rs2239185, rs7975232, rs11574129 and rs11568820	PCR	The findings indicated that the risk of OLP (oral lichen planus) was elevated in individuals possessing the rs2239185 TT genotype. Furthermore, rs2239185 and rs7975232 exhibited significant cumulative impacts on the risk of OLP.	The rs2239185 and rs7975232 variations of VDRmay affect susceptibility to OLP.
			207-Control				
4	Nigam et al. (18)	India	230-Cases	rs731236 (Taq1)	polymerase chain reaction–restriction fragment length polymorphism (PCR-RFLP)	This study finds that VDR (Taq1) polymorphism is correlated with susceptibility to oral cancer and pre-cancerous conditions in the North Indian population.	VDR polymorphism markedly diminishes the risk of oral disorders, especially the risk of leukoplakia, a form of precancerous oral lesion.
			300-Healthy controls				
5	Mohtasham et al. (10)	Iran	50-Cases	rs7975232 (Apa1)	PCR-RFLP	A notable distinction was noted in the Aa and aa genotypes relative to AA between OSCCs and controls.	A favorable correlation exists between the rs7975232 VDR polymorphism and susceptibility to OSCC.
			40-Healthy controls				

### 3.2 Meta-analysis of VDR polymorphisms on the risk of tobacco-related cancers

**Figure 5:**
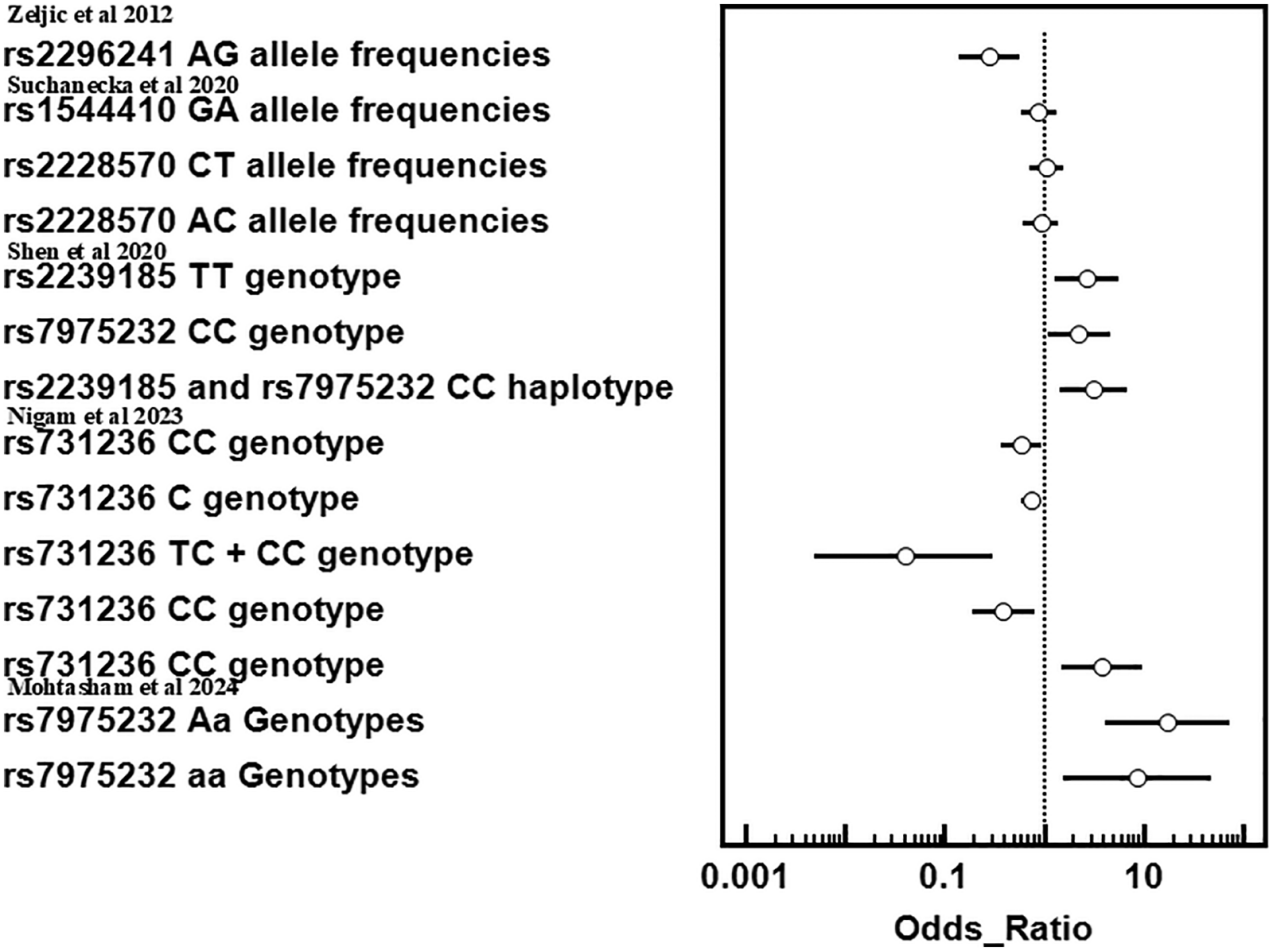
Forest plot showingVDR polymorphisms on the risk of tobacco-related cancers (*p* ≤ 0.05).

Multiple studies highlight the complex interplay between genetic polymorphisms, smoking, and aging in influencing oral health and disease risks. Zeljic et al. (2012) reported a significant reduction in oral cancer risk for individuals with the heterozygote genotype of the *CYP24A1*gene (rs2296241) compared to the wild type (OR = 0.281, *P* = 0.00001) (15). Similarly, Suchanecka et al. (2020) examined three VDR gene SNVs, with findings indicating that rs1544410 GA allele frequencies (OR = 0.8785, 95% CI = 0.5846–1.32), rs2228570 CT allele frequencies (OR = 1.041, 95% CI = 0.702–1.545), and rs2228570 AC allele frequencies (OR = 0.9231, 95% CI = 0.6237–1.366) were correlated with oral health outcomes influenced by smoking, aging, and genetic interactions (16). Shen et al.(2020) demonstrated an increased risk of oral lichen planus (OLP) for rs2239185 TT (OR = 2.68, 95% CI = 1.28–5.62, *P* = 0.009) and rs7975232 CC (OR = 2.25, 95% CI = 1.10–4.58, *P* = 0.026) (17). The rs2239185–rs7975232 CC haplotype further increased OLP risk (OR = 3.11, 95% CI = 1.42–6.83, *P* = 0.005). Conversely, Nigam et al. found the rs731236 CC genotype and C allele to significantly decrease oral disease risk (OR = 0.60, *P* = 0.04; OR = 0.75, *P* = 0.02, respectively) (18). Smokers with TC and CC genotypes exhibited decreased oral disease risk (OR = 0.04, *P* = 0.0001). However, rs731236 CC was linked to high cell differentiation (OR = 3.78, *P* = 0.008). Mohtasham et al. highlighted significant age differences between controls and OSCC patients (*P* = 0.001), with increased OSCC risk for rs7975232 Aa (OR = 17.33, 95% CI = 4.11–73.03) and aa genotypes (OR = 8.67, 95% CI = 1.55–48.49) compared to AA (Figure 5) (10).

## 4 Discussion

The results of this detailed investigation illuminate the complex interaction between variations in the VDR gene and the development of tobacco-related oral cancer. The meta-analysis of five studies highlights the complex interaction between genetic polymorphisms, smoking, aging, and oral health risks. It found a reduced oral cancer risk in individuals with the CYP24A1 heterozygote genotype. Additionally, specific VDR gene SNVs were linked to oral health outcomes, with an increased risk of oral lichen planus for certain genotypes. The rs731236 CC genotype reduced oral disease risk, while age-related variations increased OSCC risk for specific genotypes. Understanding the role of genetic predisposition, particularly VDR polymorphisms, alongside extrinsic factors like tobacco smoking, is crucial given the multifactorial origins of oral cancer (19). While tobacco smoking is the primary cause, genetic factors such as VDR polymorphisms can influence an individual's susceptibility to the disease (20), facilitating a more personalized approach to cancer risk assessment and prevention.

This review indicates a potential correlation between various VDR gene variations such as *FokI, BsmI, TaqI, and ApaI* and an increased risk of oral cancer linked to tobacco use. Several studies found a significantly higher prevalence of specific alleles, including the FokI T allele, in oral cancer patients with a history of tobacco use compared to controls (1, 21). This could be attributed to how VDR gene polymorphisms affect the expression and functionality of the vitamin D receptor, impacting cell proliferation, differentiation, and immune response, all critical in cancer progression (22).

Vitamin D plays a vital role in regulating the immune system and exhibiting anti-inflammatory and anti-carcinogenic effects. However, individuals with certain VDR variants may have a diminished ability to leverage these benefits (23). This deficiency could hinder the body's response to the carcinogenic effects of tobacco, thereby increasing the risk of developing oral cancer. However, not all studies consistently support this correlation, and discrepancies may arise from differences in study design, demographic characteristics, sample size, and environmental factors.

Tobacco consumption is known to produce reactive oxygen species (ROS) and induce chronic inflammation, both of which contribute to cancer development (24). The VDR gene is crucial for modulating the immune response to oxidative stress and inflammation. Consequently, individuals with certain VDR polymorphisms may have reduced capacity to mitigate the inflammatory and oxidative damage caused by tobacco, potentially elevating their risk for oral cancer (25).

This study aims to present evidence supporting the hypothesis that individuals with high-risk VDR genotypes may experience a synergistic effect from tobacco smoking, increasing their likelihood of developing oral cancer. The interaction between genetic predisposition and environmental carcinogens underscores the importance of considering both genetic and lifestyle factors in cancer risk assessments (26).

The five studies collectively highlight the significant role of VDR polymorphisms on the risk of tobacco-related cancers. Zeljic et al. demonstrated that individuals with the heterozygote genotype of the *CYP24A1* gene (rs2296241) had a markedly decreased risk of oral cancer (*OR* = 0.281, *P* = 0.00001), indicating a potential protective genetic effect against carcinogenesis (15). Suchanecka et al. emphasized the influence of three VDR gene SNV (rs1544410, rs2228570) on oral health, particularly in the context of smoking and aging (16). Although the odds ratios for these SNVs showed varying levels of association, they underscore the complex interplay between genetic factors, environmental exposures, and age in oral health outcomes.

Shen et al. identified specific genotypes, including rs2239185 TT and rs7975232 CC, that significantly increased the risk of OLP (*OR* = 2.68 and *OR* = 2.25, respectively) (17). Haplotype analysis further revealed that individuals carrying the CC haplotype (rs2239185-rs7975232) had an elevated OLP risk (*OR* = 3.11, *P* = 0.005), suggesting cumulative genetic effects.

In contrast, Nigam et al. highlighted protective associations for the rs731236 CC genotype and C allele, which reduced the risk of oral diseases (*OR* = 0.60–0.75, *P* < 0.05), including leukoplakia (*OR* = 0.39, *P* = 0.01) (18). Smokers with these genotypes exhibited an even greater protective effect (*OR* = 0.04, *P* = 0.0001). However, the CC genotype was linked to high-grade cellular differentiation at diagnosis, emphasizing the dual roles of genetic variants.

Lastly, Mohtasham et al. reported significant age-related differences between controls and oral squamous cell carcinoma (OSCC) patients (*P* = 0.001) and found a strong association between the rs7975232 Aa and aa genotypes and OSCC risk (*OR* = 17.33 and *OR* = 8.67, respectively) (10). These findings further support the influence of genetic predispositions in oral cancer susceptibility. These studies collectively underscore the importance of genetic polymorphisms in modulating susceptibility to oral diseases, with some variants conferring protection while others increase risk.

The potential link between VDR gene variations and tobacco-related oral cancer has significant implications for clinical practice and public health (11). Genotyping for VDR polymorphisms could help identify individuals at increased risk for oral cancer, particularly in populations with high tobacco use. This could enable the development of tailored screening programs, proactive detection strategies, and targeted therapies for high-risk patients, ultimately reducing oral cancer incidence and improving outcomes.

Additionally, the findings emphasize the need for public health initiatives that address both genetic and environmental factors in cancer prevention. Programs promoting smoking cessation, combined with strategies to enhance vitamin D levels in at-risk populations, could significantly contribute to reducing oral cancer rates.

While this review offers valuable insights into the role of VDR polymorphisms in tobacco-related oral cancer, it is essential to recognize its limitations. The variability in study designs, demographic factors, and methodologies for examining VDR polymorphisms restricts the generalizability of the findings. Furthermore, small sample sizes in many studies may have limited the statistical power to detect significant associations.

Future research should focus on conducting comprehensive, methodologically robust studies that consider potential confounding variables such as age, gender, and vitamin D levels. Investigating the interplay between VDR polymorphisms and various genetic, epigenetic, and environmental factors could lead to a more nuanced understanding of this complex relationship.

## 5 Conclusions

The findings of this research highlight the potential significance of VDR gene variations in determining the likelihood of developing oral cancer as a result of tobacco use. It is necessary to do additional research in order to evaluate these results and investigate the underlying mechanisms; despite the fact that the data suggests that there may be a correlation. There is a possibility that individualized approaches that take into account genetic predisposition, factors related to lifestyle, and vitamin D levels could offer the potential for the prevention and early identification of oral cancer, hence improving the results for patients.

## Data Availability

The raw data supporting the conclusions of this article will be made available by the authors, without undue reservation.
